# Mechanosensitive Piezo1 in Periodontal Ligament Cells Promotes Alveolar Bone Remodeling During Orthodontic Tooth Movement

**DOI:** 10.3389/fphys.2021.767136

**Published:** 2021-11-22

**Authors:** Yukun Jiang, Yuzhe Guan, Yuanchen Lan, Shuo Chen, Tiancheng Li, Shujuan Zou, Zhiai Hu, Qingsong Ye

**Affiliations:** ^1^State Key Laboratory of Oral Diseases, National Clinical Research Center for Oral Diseases, West China Hospital of Stomatology, Sichuan University, Chengdu, China; ^2^Center of Regenerative Medicine, Renmin Hospital of Wuhan University, Wuhan, China; ^3^Center of Regenerative Medicine, Massachusetts General Hospital, Harvard Medical School, Boston, MA, United States

**Keywords:** Piezo1, bone remodeling, orthodontic tooth movement, alveolar bone, mechanotransduction

## Abstract

Orthodontic tooth movement (OTM) is a process depending on the remodeling of periodontal tissues surrounding the roots. Orthodontic forces trigger the conversion of mechanical stimuli into intercellular chemical signals within periodontal ligament (PDL) cells, activating alveolar bone remodeling, and thereby, initiating OTM. Recently, the mechanosensitive ion channel Piezo1 has been found to play pivotal roles in the different types of human cells by transforming external physical stimuli into intercellular chemical signals. However, the function of Piezo1 during the mechanotransduction process of PDL cells has rarely been reported. Herein, we established a rat OTM model to study the potential role of Piezo1 during the mechanotransduction process of PDL cells and investigate its effects on the tension side of alveolar bone remodeling. A total of 60 male Sprague-Dawley rats were randomly assigned into three groups: the OTM + inhibitor (INH) group, the OTM group, and the control (CON) group. Nickel-titanium orthodontic springs were applied to trigger tooth movement. Mice were sacrificed on days 0, 3, 7, and 14 after orthodontic movement for the radiographic, histological, immunohistochemical, and molecular biological analyses. Our results revealed that the Piezo1 channel was activated by orthodontic force and mainly expressed in the PDL cells during the whole tooth movement period. The activation of the Piezo1 channel was essential for maintaining the rate of orthodontic tooth movement and facilitation of new alveolar bone formation on the tension side. Reduced osteogenesis-associated transcription factors such as Runt-related transcription factor 2 (RUNX2), Osterix (OSX), and receptor activator of nuclear factor-kappa B ligand (RANKL)/osteoprotegerin (OPG) ratio were examined when the function of Piezo1 was inhibited. In summary, Piezo1 plays a critical role in mediating both the osteogenesis and osteoclastic activities on the tension side during OTM.

## Introduction

Induced by mechanical loading, orthodontic tooth movement (OTM) is a process depending on the remodeling of periodontal tissues (mainly alveolar bone, periodontal ligament, and cementum) surrounding the roots (Kumar et al., 2015). During tooth movement, alveolar bone remodeling is triggered by biologic responses of periodontal ligament (PDL), with bone formation taking place on the tension side and resorption occurring on the compression side (Vansant et al., 2018). The association between the mechanical stimuli and periodontal ligament has been demonstrated by lots of previous studies from the viewpoints of both cellular biology and signaling molecules.

Periodontal ligament cells play crucial roles in maintaining periodontal homeostasis and regulating periodontal tissue remodeling. PDL cells have multiple functions locally including forming and maintaining periodontal ligaments, mediating alveolar bone and cementum repair, and periodontal tissue regeneration (Liu et al., 2018; Maeda, 2020). According to recent studies, PDL cells can sense mechanical stress, transform mechanical stimuli into biochemical signals, and eventually regulate alveolar bone remodeling by secreting multiple factors (Yang et al., 2018). For example, tissue plasminogen activator (tPA) and plasminogen activator inhibitor-1 (PAI-1), matrix metalloproteinases (MMPs) and their inhibitors, and cytokines including prostaglandin 2 (PGE2) and interleukin-6 (IL-6) are secreted under mechanical stimuli to regulate periodontal tissues remodeling (Römer et al., 2013; Schara et al., 2013; Wu et al., 2015), whereas receptor activator of nuclear factor-kappa B ligand (RANKL), RANK, and osteoprotegerin (OPG) system are reported to be involved in the regulation of osteoclast differentiation by PDL cells (Grant et al., 2013; Feng et al., 2018). However, during OTM, the specific mechanism by which PDL cells perceive the mechanical stimulus, transfer it into biological signals, and ultimately contribute to alveolar bone remodeling remains unclear.

Recently, the ion channels on the surface of PDL cells involved in mechanotransduction pathways have been proposed as a promising new research direction to this problem (Sachs, 2015; Jin S.S. et al., 2020). The Piezo family of non-selective cationic channels was first identified as pore-forming subunits of excitatory mechanosensitive ion channels permeable to Na^+^, K^+^, and Ca^2+^ in 2010 (Coste et al., 2010). The mammalian Piezo channel is a three-bladed, propeller-shaped trimeric complex containing over 2,500 amino acids, which includes two subtypes, Piezo1 and Piezo2 (Wang and Xiao, 2018). Piezo1 is predominantly expressed in non-sensory tissues exposed to mechanical force, while Piezo2 is primarily expressed in sensory tissues (Wu et al., 2017). The Piezo1 channel has been implicated in bone remodeling by growing literature. Several *in vivo* studies have provided evidence that Piezo1 removed from mice osteoblastic cells caused bone loss and spontaneous fractures with increased bone resorption, suggesting the possible role of Piezo1 in regulating mechanical load-dependent bone formation and remodeling (Li et al., 2019; Wang et al., 2020b; Zhou et al., 2020). Piezo1 is also expressed in mesenchymal stem cells (MSCs) as a factor for cell fate determination of MSCs under hydrostatic pressure, which also implies the relevance of Piezo1 and bone formation (Sugimoto et al., 2017).

Piezo1 also exists on the membrane of primary PDL cells (Jin et al., 2015). *In vitro* studies have confirmed that the Piezo1 ion channel can transmit mechanical signals and regulate both the osteogenic differentiation and osteoclastogenesis of PDL cells *via* various signaling including extracellular regulated protein kinases (ERK), nuclear factor-kappa B (NF-κB), and Notch1 signaling pathways (Jin et al., 2015; Shen et al., 2020; Wang et al., 2020a). These results suggest the possible mechanotransduction role of Piezo1 in the process of OTM. Till now, the functional relevance of Piezo1 and alveolar bone remodeling on the tension side remains unclear.

This study investigated the hypothesis that Piezo1 might function as a critical mechanotransducer that mediates mechanotransduction process of PDL cells, consequently determining alveolar bone remodeling. Firstly, the expression patterns of the Piezo1 channel during OTM were explored in a rat model on the tension side. Then, the mechanotransduction role of the Piezo1 channel in alveolar bone remodeling was verified via local periodontal inhibition of Piezo1 channel activity.

## Materials and Methods

### Establishment of Animal Models

The Research Ethics Committee approved all the animal experiments of the State Key Laboratory of Oral Diseases in Chengdu, China (WCHSIRB-D-2017-181). A total of 72 male Sprague-Dawley rats aged 8 weeks (weighing 200 ± 10 g) were obtained from the Experimental Animal Center of the Sichuan University. Then, the animals were randomly divided into three groups of 24 animals each: the OTM + inhibitor (INH) group, the OTM group, and the control (CON) group. The rats in the OTM and OTM + INH groups received orthodontic appliances and the CON group did not receive the appliance. The OTM animal model was established as described previously (Li et al., 2013). A nickel-titanium coil spring (3M Unitek, Monrovia, CA, United States) was fixed between the maxillary left first molar and the incisors to generate a force of 40 g. The appliances were activated immediately upon insertion and the fit was checked daily. No reactivation was performed during the experimental period. The animals in the OTM + INH group received a subcutaneous injection of Piezo1 inhibitor Grammostola spatulata mechanotoxin 4 (GsMTx4) (Abcam, San Francisco, CA, United States) at a dosage of 20 μl with a concentration of 10 μM every other day, whereas the OTM and CON groups received only the vehicle. A total of six rats of each group were killed immediately on day 0 (the day before orthodontic force application) to serve as negative controls and on days 3, 7, and 14 after tooth movement. Alveolar bone blocks included the left first molar, which was harvested for further analysis.

### Micro-CT Analysis

The samples were fixed in 4% paraformaldehyde solution for 24 h and scanned by using a high-resolution Micro-CT50 system (Scanco Medical, Wangen-Brüttisellen, Switzerland). Scanning of the specimens was carried out at 70 kV and 114 mA with an integration time of 500 ms and a voxel resolution of 10 μm. The amount of OTM was measured by the spacing between the cementum–enamel junction (CEJ) levels of the first and second left molars. In this study, a 200 μm × 200 μm × 600 μm cube of trabecular bone distal to the middle part of the distal buccal root of the maxillary left first molar was selected as the region of interest for analysis. The distance between the cube and the root was 100 μm. Then, parameters including the bone volume/total volume (BV/TV) ratio, trabecular spacing (Tb.Sp), trabecular number (Tb.N), and trabecular thickness (Tb.Th) were calculated at days 3, 7, and 14 after the OTM.

### Histological Staining

After micro-CT scanning, the left half of the maxilla of each animal was decalcified in 14% ethylenediaminetetraacetic acid (EDTA) (pH 7.4) for a month. Then, all the specimens were dehydrated in a series of alcohol baths and embedded in paraffin. Next, the samples, including the maxillary molars, were excised into 5 μm thick frontal sections in the sagittal direction. H&E staining (G1120; Solarbio, Beijing, China), tartrate-resistant acid phosphatase (TRAP) staining (Sigma-Aldrich, St. Louis, MO, United States), and Masson’s trichrome staining (G1340; Solarbio, Beijing, China) were performed for the histological analyses. Multinucleated cells adjacent to the tension side of the periodontal area were counted as TRAP-positive osteoclasts. Two independent investigators counted the number of TRAP-positive cells. Masson’s trichrome staining was used to identify the collagen fiber arrangement on the tension side.

### Immunofluorescence Staining

Tissue sections were heated at 95°C for 30 min for antigen retrieval, washed with phosphate-buffered saline (PBS) for 5 min, and blocked in 10% goat serum for 30 min at 37°C. Then, the samples were incubated with rabbit anti-Piezo1 (Abcam, Cambridge, MA, United States, 1:200) overnight at 4°C. On the following day, slides were incubated with the Alexa Fluor 488 goat anti-rabbit (ZF-0511, 1:200, Zhongshan Bio-Tech, Beijing, China) for 1 h at 37°C and counterstained with 4’,6-diamidino-2-phenylindole (DAPI) (Sigma, St. Louis, MO, United States) for 15 min. After washing with PBS, stained sections were observed by using fluorescent microscopy (DMI 6000; Leica, Wetzlar, Germany). Quantitative analyses of the images were done by the Image-Pro Plus 6.0 Software (Media Cybernetics, Bethesda, MD, United States).

### Immunohistochemical Staining

Immunohistochemical staining was performed as described previously (Li et al., 2021). Sections were incubated with primary antibodies diluted in blocking solution with different optimized dilution rates: OPG (dilution 1:100; R1608-4), Runt-related transcription factor 2 (RUNX2) (dilution 1:100; ET1612-47), Osterix (OSX) (dilution 1:400; ER1914-47), collagen type 1 (COL1) (dilution 1:100; ET1609-68), and alkaline phosphatase (ALP) (dilution 1:100; ET1601-21) from Huabio (Hangzhou, China); RANKL (dilution 1:200, ab169966) from Abcam (Shanghai, China). After rinsing, the slides were incubated with goat anti-rabbit immunoglobulin G (IgG) secondary antibody horseradish peroxidase (HRP) conjugated (SP-9001, Zhongshan Bio-Tech, Beijing, China) for 30 min at 37°C. The immune reaction was visualized by using a 3,3′-diaminobenzidine (DAB) Kit (ZLI-9017, Zhongshan Bio-Tech, Beijing, China) according to the instructions of the manufacturers. Slides were counterstained with H&E and viewed by using a light microscope (Nikon Eclipse 80i microscope, Tokyo, Japan). The means of integrated optical density (IOD) of IHC staining was analyzed by the Image-Pro Plus 6.0 Software (Media Cybernetics, Bethesda, MD, United States).

### Western Blotting

Total proteins from all three groups were extracted from fresh tissue on day 3 and day 7 by using the Minute™ Total Protein Extraction Kit for Bone Tissue (Invent Biotechnologies Incorporation, Eden Prairie, MN, United States) according to the instructions of the manufacturer. 50 μg of proteins from each sample were loaded on sodium dodecyl sulfate (SDS)–polyacrylamide gel electrophoresis (PAGE) and electrotransferred to polyvinylidene difluoride (PVDF) membranes (Invitrogen, Carlsbad, CA, United States). The membranes were blocked with 5% bovine serum albumin (BSA) and incubated with primary antibodies overnight. The following antibodies were used in this study to examine the protein levels: RANKL (dilution 1:1000, ab239607) from the Abcam (Shanghai, China); OPG (dilution 1:1000; R1608-4), RUNX2 (dilution 1:1000; ET1612-47), OSX (dilution 1:1000; ER1914-47), COL1 (dilution 1:1000; ET1609-68), and ALP (dilution 1:1000; ET1601-21) from the Huabio (Hangzhou, China). On the next day, blots were incubated with horseradish peroxidase conjugated with anti-rabbit IgG secondary antibodies (dilution 1:500; Thermo Pierce, Rockford, IL, United States) and detected by using an enhanced chemiluminescence substrate kit (Applygen, Beijing, China). The intensity of each band was quantified by using the Image-Pro Plus 6.0 Software (Media Cybernetics, Bethesda, MD, United States) and normalized to glyceraldehyde-3-phosphate dehydrogenase (GAPDH) values.

### Statistical Analysis

The GraphPad Prism Software version 7 (GraphPad, San Diego, CA, United States) was used for statistical analysis. Data were expressed as the mean ± SD from at least three independent experiments. The statistical difference among the three groups was analyzed by the one-way ANOVA analysis followed by the Student-Newman-Keuls (SNK) methods of analysis of variance. *p* < 0.05 was considered statistically significant.

## Results

### Piezo1 Channel Was Activated by Orthodontic Force on the Tension Side During Orthodontic Tooth Movement

**FIGURE 1:**
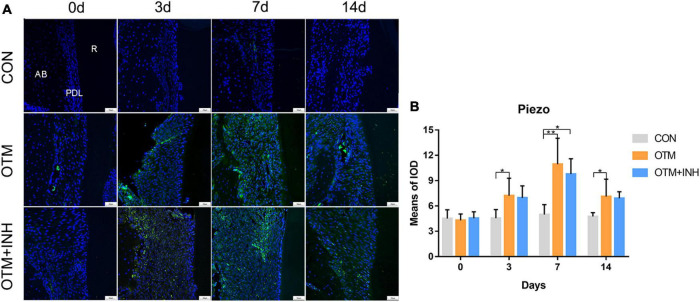
The expression of Piezo1 was activated by orthodontic force on the tension side of distobuccal roots. **(A)** Immunofluorescence staining showed that Piezo1 was mainly expressed in the PDL of the OTM and OTM + INH groups after orthodontic force application. **(B)** The means of the fluorescent intensity of Piezo1. The expression of Piezo1 in the OTM group was significantly greater than that in the CON group from day 3 to day 14. Data are presented as the mean ± SD. *p < 0.05. **p < 0.01. Scale bar = 50 μm. INH, inhibitor; OTM: orthodontic tooth movement; AB, alveolar bone; PDL, periodontal ligament; R, root.

To test our hypothesis that the Piezo1 channel is a crucial mechanosensor required for alveolar bone remodeling, the expression patterns of Piezo1 within the periodontal area on the tension side were first examined by IF staining. Our results showed that Piezo1 was mainly expressed in the PDL, while it is rarely found in the alveolar bone. Specifically, the Piezo1 expressed faintly in the PDL of the CON group, while activated by orthodontic force application in the OTM group during the entire experimental period (Figure 1). Semi-quantitative analysis revealed that the expression of Piezo1 in the OTM group started to increase on day 3, reached its peak after 7 days of tooth movement, and only declined on day 14 compared to the CON group. When Piezo1 channel inhibitor GsMTx4 was applied, no significant change of the expression of Piezo1 was found in the OTM + INH group. These findings indicated that the expression of Piezo1 was activated by orthodontic force in the PDL during OTM.

### Inhibition of Piezo1 Channel Hinders Orthodontic Tooth Movement and Decreases Bone Mass

**FIGURE 2:**
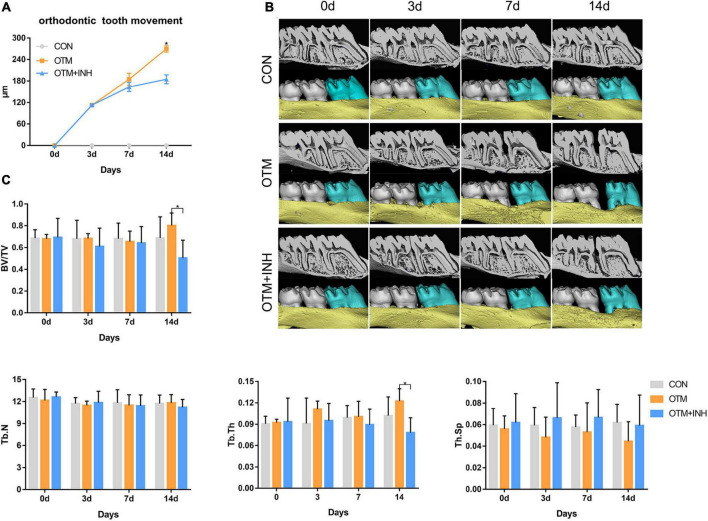
Inhibition of Piezo1 hindered the distance of OTM and decreased bone mass. **(A)** Micro-CT and three-dimensional (3D) reconstruction images showed the distance of OTM. **(B)** The distance of OTM from day 0 to day 14. The distance was increased by orthodontic force overtime in the OTM group while declined by GsMTx4 in the OTM + INH group. *p < 0.05, the OTM vs. OTM + INH groups. **(C)** Parameters including bone volume/total volume (BV/TV), trabecular number (Tb.N), trabecular thickness (Tb.Th), and trabecular spacing (Tb.Sp) were examined from day 0 to day 14. BV/TV and Tb.Th of the OTM group were significantly greater than that of the OTM + INH and control (CON) groups on day 14 of OTM. *p < 0.05. Data are presented as the mean ± SD.

To confirm the effect of the Piezo1 channel on OTM, the Piezo1 inhibitor GsMTx4 was adopted in this study to inactivate the Piezo1 channel. Under normal circumstances, the distance between the first and second molars was increased over time with a relatively rapid increment on the late-stage (day 7 to day 14). When the Piezo1 channel was inactivated by GsMTx4, the distance of tooth movement was significantly decreased from day 7 to day 14 (Figures 2A,B). These results suggested that the proper function of the Piezo1 channel was necessary for achieving the usual distance and rate of OTM.

Next, to evaluate the alveolar bone mass and microarchitectural changes of alveolar bone in the tension area of all the three groups, the parameters of BV/TV, Tb.N, Tb.Sp, and Tb.Th were examined through micro-CT analysis. As shown in Figure 2C, all the parameters showed stable expression levels in the CON group with no statistical difference throughout the experiment. When all the three groups were compared together, we found that BV/TV and Tb.Th in the tension area of the OTM group were significantly higher than that in the OTM + INH group and CON group on day 14 of OTM. No significant difference in values for Tb.N and trabecular spacing (Th.Sp) was found among the three groups. The above results showed that alveolar bone mass was decreased by inhibiting the Piezo1 channel during OTM.

### Histological Changes of Periodontal Tissues During Orthodontic Tooth Movement

**FIGURE 3:**
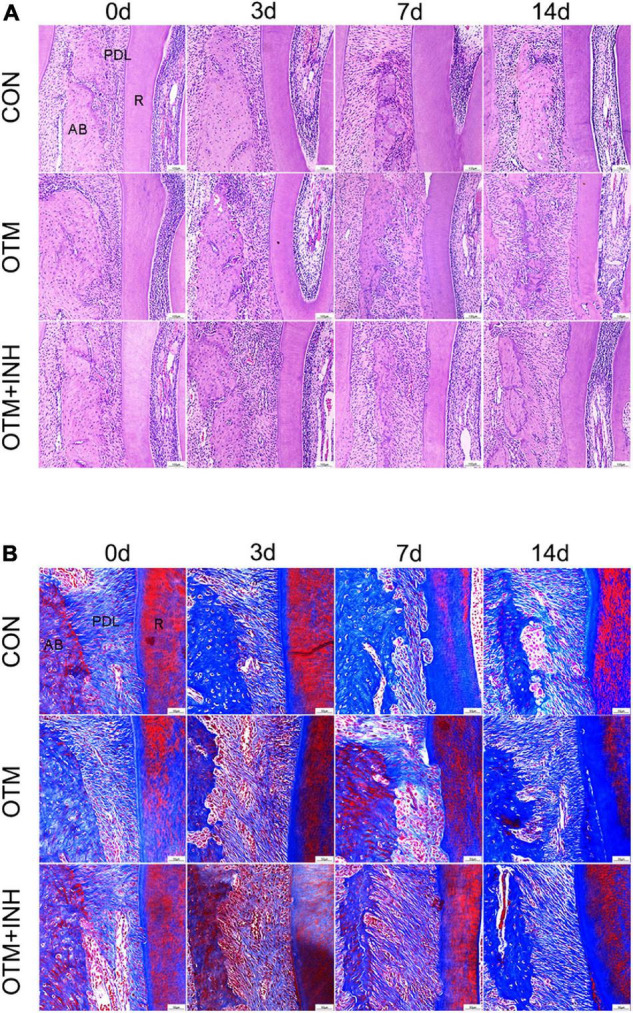
Histological changes of PDL on the tension side of the distobuccal roots. HE staining **(A)** and Masson’s trichrome staining **(B)** of PDL. Periodontal fibers were stretched by applying orthodontic force in the OTM group, while remodeling was delayed by the inhibitor of Piezo1 in the OTM + INH group. Scale bar for **(A)** = 100 μm. Scale bar for **(B)** = 50 μm. AB, alveolar bone; R, root.

Histological changes of PDL cells were examined by using the H&E and Masson’s trichrome staining. On day 0, periodontal fibers showed a meshwork arrangement and each PDL fiber showed a wavy configuration in the PDL in all three groups. After 3 days of orthodontic movement, periodontal ligament space gradually increased in the OTM and OTM + INH groups. In both the OTM and OTM + INH groups, the typical wavy arrangement of the periodontal fibers was lost and the periodontal fibers were stretched between the bone and the root. On day 7, periodontal ligament fibers appeared to be relaxed in the OTM group compared to day 3, while fully stretched in the OTM + INH group. On day 14, fiber arrangement in the OTM groups recovered to the wavy configuration before OTM, while slightly stretched in the OTM + INH group. At this time, periodontal ligament fibers on the tension side were arranged again in all three groups (Figures 3A,B).

### Piezo1 Channel Is Critical for Enhanced Expression of Osteogenesis-Related Factors on the Tension Side During Orthodontic Tooth Movement

**FIGURE 4:**
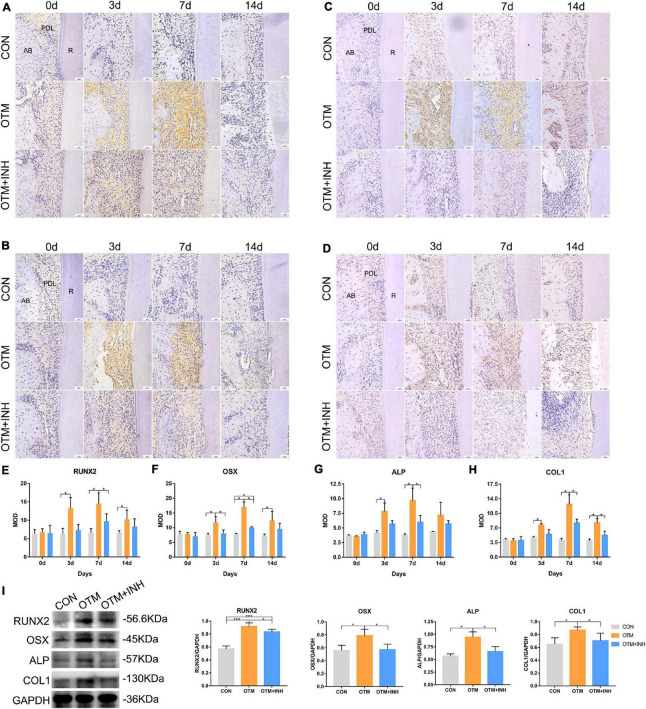
Inhibition of Piezo1 channel suppressed expression of osteogenesis-related factors during OTM on the tension side. Immunohistochemical staining of RUNX2 **(A)**, OSX **(B)**, ALP **(C)**, and COL1 **(D)** of PDL on the tension side of the distobuccal roots and the means of the integrated optical density (IOD) of RUNX2 **(E)**, OSX **(F)**, ALP **(G)**, and COL1 **(H)**. The expression of RUNX2, OSX, ALP, and COL1 in the OTM group increased significantly on days 3, 7, and 14 compared with that of the CON group, while it was significantly reduced by GsMTx4 on day 3 and day 7 for OSX, day 7 for RUNX2 and ALP, and day 7 and day 14 for COL1. **(I)** Western blot analyses were carried out to detect the protein levels of RUNX2, OSX, ALP, and COL1 on day 7 of OTM. GsMTx4 significantly reduced the protein expression of RUNX2, OSX, ALP, and COL1 on day 7 in the OTM + INH group. Data are presented as the mean ± SD. *p < 0.05, ***p < 0.001. Scale bar = 50 μm. RUNX2, Runt-related transcription factor 2; OSX, Osterix; ALP, alkaline phosphatase; COL1, collagen type 1; AB, alveolar bone; R, root.

Immunohistochemical expression of osteogenesis-related factors was examined to evaluate the further correlation between Piezo1 and osteogenesis (Figures 4A–D). After applying orthodontic force, the expression of RUNX2, OSX, ALP, and COL1 in the OTM group was all significantly increased on days 3, 7, and 14 compared with the CON group. All the factors reached peak expression in the OTM group on day 7 (with over 2-fold greater OD values for RUNX2 and OSX and 3-fold greater OD values for ALP and COL1) compared with those in the CON group. When Piezo1 was inhibited by GsMTx4, the expression of these osteogenesis-related factors was all decreased in the OTM + INH group. The expression of RUNX2 and ALP of the OTM + INH group was significantly lower than the OTM group on day 7. For OSX, its expression in the OTM + INH group was significantly lower than the OTM group on day 3 and day 7. For COL1, the OTM + INH group was significantly lower than the OTM group on day 7 and day 14 (Figures 4E–H). Consistent with IHC staining, Western Blot (WB) results showed that protein levels of osteogenesis factors on day 7 were all reduced by GsMTx4 in the OTM + INH group, which indicated that the Piezo1 channel was critical for the expression of osteogenesis-related factors on the tension side during OTM (Figure 4I).

### Inhibition of Piezo1 Channel Suppressed Osteoclastic Activities on the Tension Side During Orthodontic Tooth Movement

**FIGURE 5:**
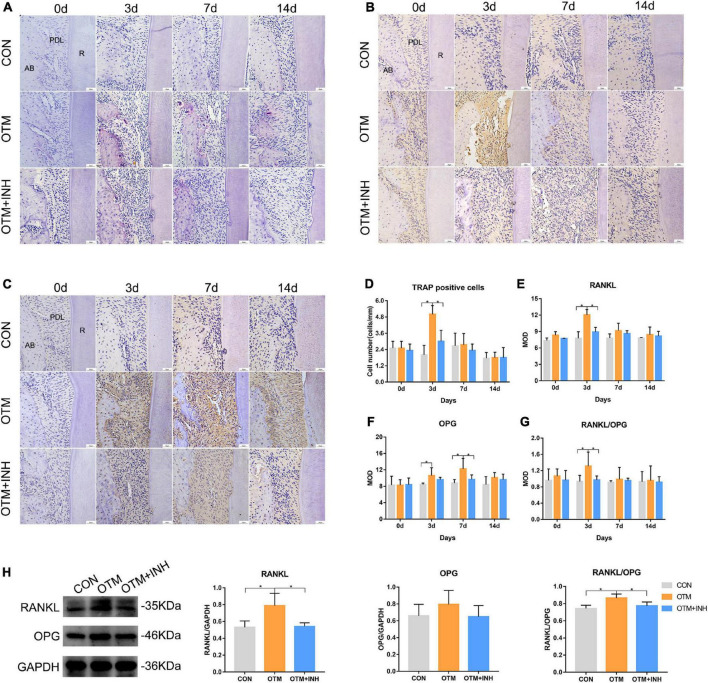
Inhibition of Piezo1 suppressed osteoclastic activities on the tension side. TRAP staining **(A)** and quantification of TRAP-positive cells **(D)**. TRAP-positive osteoclasts were detected near the tension side of the roots. On day 3, the number of osteoclasts in the OTM group was significantly greater than that of the CON and the OTM + INH groups. Scale bar = 50 μm. Immunohistochemical staining of RANKL **(B)** and OPG **(C)** of PDL on the tension side of the distobuccal roots. The means of the integrated optical density (IOD) of RANKL **(E)**, OPG **(F)**, and RANKL/OPG ratio **(G)** were shown. On day 3, the RANKL/OPG ratio of the OTM group was significantly greater than that of the CON and OTM + INH groups. Western blot analyses **(H)** were carried out to detect the protein levels of RANKL and OPG on day 3 of OTM. The protein expression of the RANKL/OPG ratio increased significantly in the OTM group and was reduced by GsMTx4 in the OTM + INH group. Data are presented as the mean ± SD. *p < 0.05. Scale bar = 50 μm. TRAP, tartrate-resistant acid phosphatase; RANKL, receptor activator of nuclear factor-kappa B ligand; OPG, osteoprotegerin; AB, alveolar bone; R: root.

The osteoclastic activities were also investigated on the tension side during OTM. TRAP staining was performed to observe the proportion of TRAP-positive cells among the three groups. On day 3, a remarkable increase in the number of osteoclasts was observed in the OTM group which is significantly higher than the OTM + INH and CON groups (*p* < 0.05). On days 7 and 14, only a few TRAP-positive cells were observed and no significant difference was found among the three groups (Figures 5A,D). Then, the expression of RANKL and OPG was evaluated. The immunoreactivity of RANKL was mainly observed in the osteoblasts and osteoclast-like cells on the surface of alveolar bone with strongly positive staining in the cytoplasm and weakly positive staining in the extracellular matrix of the periodontal ligament area. The expression level of RANKL in the OTM group was significantly higher than the OTM + INH and CON groups on day 3 and declined after that. No significant change was found among all the three groups on days 7 and 14 (Figures 5B,E). The immunoreactivity of OPG was mainly observed in some osteoblasts with strongly positive staining in the cytoplasm and weakly positive staining in the extracellular matrix of the periodontal ligament. In this study, the expression of OPG in the OTM group was gradually increased from day 3, peaked on day 7, and decreased slightly after that. With respect to the CON group, the OPG expression in the OTM group was significantly higher on day 3. On day 7, the OPG expression in the OTM group was significantly higher than in the OTM + INH and CON groups (Figures 5C,F). The RANKL/OPG increased rapidly in the OTM group and was significantly greater than OTM + INH and CON groups on day 3 (Figure 5G). On days 7 and 14, no significant change was found in the ratio of RANKL/OPG among all three groups. On day 3, the WB results of RANKL/OPG were consistent with the IHC staining (Figure 5H).

## Discussion

Alveolar bone and PDL undertake a mechanical loading-induced remodeling process during OTM. However, it remains unclear how the alveolar bone and PDL sense mechanical force. Piezo1, a non-selective cationic channel, responds to various forms of mechanical stimulation including poking, stretching, shear stress, substrate deflection, and mediates Ca^2+^ influx upon opening to initiate downstream Ca^2+^ signaling (Sun et al., 2019). Recently, the engagement of Piezo1 in bone remodeling has been reported by an increasing number of studies (Li et al., 2019; Sun et al., 2019). To reveal the possible mechanotransduction role of Piezo1 in the process of OTM, this study focused on characterizing the expression and function of the mechanically activated Piezo1 channel of PDL cells on alveolar bone remodeling.

In this study, the localization of IF results showed that Piezo1 was mainly expressed on PDL cells and located in the plasma membrane and nucleus. The expression of Piezo1 in the OTM group was upgraded during the application of orthodontic force. Similar results recently reported that mechanical stress is associated with increased Piezo1 activation in PDL cells (Jin et al., 2015; Shen et al., 2020; Wang et al., 2020a). Therefore, we speculated that the Piezo1 channel is a crucial mechanosensor through which the PDL senses and translates the orthodontic force into a biological response, which further influences the alveolar bone remodeling.

To verify our assumption concerning the regulation effect of the Piezo1 channel, we administered the Piezo1 inhibitor GsMTx4 to orthodontically loaded rats as the OTM + INH group. No significant change in the expression of Piezo1 was found in the OTM + INH group when GsMTx4 was applied. This observation can be explained as follows. As a Piezo1-specific inhibitor, GsMTx4 acts by perturbing the interface between the Piezo1 channel and the lipid bilayer, which selectively blocks the gating of this cation-selective channel (Gnanasambandam et al., 2017; Suchyna, 2017). In other words, the GsMTx4 blocks the function of the Piezo1 channel without interfering with its expression and our result was consistent with its mechanism of action. In fact, GsMTx4 significantly reduced OTM in both the distance and rate and delayed the fiber remodeling. Micro-CT revealed that BV/TV and Tb.Th in the OTM + INH group tension area was significantly lower than in the OTM group, especially on day 14 of OTM. Taken together, this part of the experiment revealed that the inhibition of the Piezo1 channel by GsMTx4 local injection hinders OTM and new bone formation on the tension side.

Meanwhile, it is interesting to note that although the Piezo1 channel was greatly inhibited by GsMTx4, the OTM was not completely ceased in the OTM + INH group. Generally speaking, various mechanosensors, including ion channels, function synergistically during OTM (Guo et al., 2019; Du et al., 2020; Jin P. et al., 2020). We speculated that the biological communication between other mechanosensors and their corresponding downstream molecules might be responsible for the OTM in the OTM + INH group.

Since alveolar bone remodeling during OTM is a constant balanced coupling reaction by bone-resorbing osteoclasts and bone-forming osteoblasts working together, we further investigated the regulatory effects of Piezo1 on the downstream osteoblastic- and osteoclastic-related biomarkers. Zhou et al. (2020) reported mechanosensitive channels Piezo1 as critical force sensors required for bone development and osteoblast differentiation. In mesenchymal or osteoblast progenitor cells, loss of Piezo1 inhibited osteoblast differentiation and increased bone resorption, leading to multiple spontaneous bone fractures in newborn mice (Zhou et al., 2020). As described in the results, this study found that inhibition of the Piezo1 channel inhibited osteogenic activity (Li et al., 2019). ALP is commonly considered an early bone marker and COL1, a major component of the new bone matrix, is considered a midmarker (Saleh et al., 2018). Together with critical osteogenesis-associated transcription factors RUNX2 and OSX, they were adopted in this study to investigate the osteogenic activities on the tension side. IHC staining showed that RUNX2, OSX, ALP, and COL1 expression levels increased with time, which indicated active osteogenesis on the tension side of alveolar bone under mechanical force. When the Piezo1 channel was blocked by inhibitor GsMTx4, the expression of RUNX2, OSX, ALP, and COL1 were all significantly decreased. Western blot analysis was also adopted in this study to determine the protein levels of these osteogenesis-related factors on the tension side of alveolar bone. As expected, western blot results showed a significant decrease in the protein expression level of RUNX2, OSX, ALP, and COL1 in the OTM + INH group compared with the OTM group. Previous studies showed that conditional deletion of Piezo1 in the osteoblasts and osteocytes notably reduced bone mass and strength in mice (Li et al., 2019; Zhou et al., 2020). Conversely, administration of a Piezo1 agonist to adult mice increased bone mass, mimicking the effects of mechanical loading. Our results were consistent with these previous studies. Taken together, these results suggested Piezo1 channel was essential for the regulation of the normal osteogenesis process of alveolar bone remodeling on the tension side.

The number of TRAP-positive cells and the ratio of RANKL/OPG were adopted in this study to reflect the osteoclastic activities during OTM. In this study, the number of TRAP-positive cells increased on day 3 in the OTM group and was significantly higher than the other two groups. The expression levels of RANKL and OPG were determined by both IHC staining and Western blot to reflect the role of the Piezo1 channel in the osteoclastic activities. Both methods showed consistent results that RANKL/OPG was more significant in the OTM group than the other two groups. It was demonstrated that Piezo1 might have a vital transduction role in osteoclastogenesis. Piezo1, in osteoblastic cells, in response to mechanical loads, controls the yes-associated protein (YAP)-dependent expression of type II and IX collagens, which regulate osteoclast differentiation (Wang et al., 2020b). Moreover, Jin et al. (2015) found that Piezo1 inhibitor GsMTx4 repressed osteoclastogenesis in the mechanical stress pretreated PDLCs-RAW264.7 coculture system *in vitro*. Our findings were consistent with these previous studies.

Taken together, our investigation demonstrated that the Piezo1 channel played a critical role in mediating both the osteogenesis and osteoclastic activities, and thus, promoting bone remodeling during OTM. By inducing the expression of critical osteogenesis-associated transcription factors such as RUNX2 and OSX, the Piezo1 channel facilitated new alveolar bone formation on the tension side. The activated expression level of the Piezo1 channel may be essential for maintaining the regular rate of OTM and may contribute to the stability of bone remodeling in the late phase of tooth movement. The specific molecular mechanisms behind the Piezo1 pathway on alveolar bone remodeling are worthy of being further elucidated.

## Conclusion

In summary, this study first reveals the expression patterns of the Piezo1 channel on the tension side during OTM. The Piezo1 channel plays a vital role in bone remodeling on the tension side, promoting osteogenesis and osteoclastic activities. Via essential mechanotransduction approaches, the activation of the Piezo1 channel is essential for maintaining the OTM and alveolar bone homeostasis. The conclusion of this study may provide a theoretical basis for therapeutic applications of local regulation of the Piezo1 channel, which represents an intriguing target for inducing stable and controlled accelerated alveolar bone remodeling during tooth movement.

## Data Availability Statement

The raw data supporting the conclusions of this article will be made available by the authors, without undue reservation.

## Ethics Statement

The animal study was reviewed and approved by the Research Ethics Committee of State Key Laboratory of Oral Diseases in Chengdu.

## Author Contributions

YJ, SZ, and ZH designed the experiments. YJ, YG, YL, and TL performed the *in vivo* experiments. YJ, YG, and SC collected and analyzed the *in vivo* experiments. YJ, TL, QY, and ZH analyzed and confirmed all the data and prepared the manuscript. SZ, QY, and ZH made final approval of the manuscript. All authors reviewed the manuscript.

## Conflict of Interest

The authors declare that the research was conducted in the absence of any commercial or financial relationships that could be construed as a potential conflict of interest.

## Publisher’s Note

All claims expressed in this article are solely those of the authors and do not necessarily represent those of their affiliated organizations, or those of the publisher, the editors and the reviewers. Any product that may be evaluated in this article, or claim that may be made by its manufacturer, is not guaranteed or endorsed by the publisher.
